# Hypertriglyceridemia-waist and risk of developing type 2 diabetes: The Rural Chinese Cohort Study

**DOI:** 10.1038/s41598-017-09136-x

**Published:** 2017-08-22

**Authors:** Yongcheng Ren, Yu Liu, Xizhuo Sun, Kunpeng Deng, Chongjian Wang, Linlin Li, Lu Zhang, Bingyuan Wang, Yang Zhao, Junmei Zhou, Chengyi Han, Hongyan Zhang, Xiangyu Yang, Xinping Luo, Chao Pang, Lei Yin, Tianping Feng, Jingzhi Zhao, Ming Zhang, Dongsheng Hu

**Affiliations:** 1 Department of Epidemiology and Health Statistics, College of Public Health, Zhengzhou University, Zhengzhou, Henan People’s Republic of China; 2 The Affiliated Luohu Hospital of Shenzhen University Health Sciences Center, Shenzhen, Guangdong, People’s Republic of China; 3 Yantian Entry-exit Inspection and Quarantine Bureau, Shenzhen, Guangdong, People’s Republic of China; 4 Department of Preventive Medicine, Shenzhen University Health Sciences Center, Shenzhen, Guangdong, People’s Republic of China; 5 Department of Prevention and Health Care, Military Hospital of Henan Province, Zhengzhou, Henan People’s Republic of China

## Abstract

Limited information is available on the effect of hypertriglyceridemia–waist (HTGW) combination and its dynamic status on the risk of type 2 diabetes mellitus (T2DM) in rural China. A cohort of 12,086 participants 18 to 92 years old was retained in this study. Kaplan-Meier analysis yielded the cumulative incidence of T2DM for each phenotype group (based on TG level and waist circumference [WC] at baseline). Cox regression yielded hazard ratios relating HTGW (based on TG level and WC at baseline and follow-up) to risk of developing T2DM. After a median follow-up of 6.0 years (71,432 person-years of follow-up), T2DM developed in 621 participants. For HTGW participants, the incidence of T2DM was 26.4/1000, 20.6/1000, and 21.9/1000 person-years for males, females, and overall, respectively. The adjusted HR for HTGW associated with T2DM was 7.63 (95% CI 4.32–13.49) for males and 7.75 (4.71–12.78) for females. Compared with consistent HTGW, with transformation from baseline HTGW to normal WC and normal triglycerides level at follow-up, the risk of developing T2DM was reduced by 75% and 78% for males and females. HTGW is a major risk factor for T2DM, but the risk could be reduced by improved triglycerides level and WC.

## Introduction

The prevalence of the hypertriglyceridemia-waist (HTGW) phenotype is increasing substantially over time. The third National Health and Nutrition Examination Survey (NHANES III), a probability sample of the non-institutionalized civilian US population studied in 1988–1994 (4448 males, 4735 females), reported an HTGW prevalence of more than 24.8%^1^. In 1999, among participants from a European cohort representative of a contemporary Western population (9506 males, 12,281 females), the HTGW prevalence was 29.1% (males 31.2%, females 27.5%)^2^. The Tehran Lipid and Glucose Study (TLGS), a representative sample of residents in district No. 13 of Tehran (3004 males and 3830 females), found an HTGW prevalence of 32.7% (males 41.5%, females 25.8%) in 2009^3^. In 2005, the Nutrition and Health of Ageing Population in China Study of non-institutionalized people in Beijing and Shanghai (1458 males, 1831 females) reported an HTGW prevalence of 16.9% (males 14.3%, females 19.1%)^4^. Furthermore, the occurrence of HTGW had a youth-oriented tendency^5–7^: a population-based cross-sectional study of non-institutionalized people aged 13–15 years in Qin Huang Dao, China (846 males, 819 females) found an HTGW prevalence of 7.0% (males 6.9%, females 7.2%) in 2010^8^. HTGW has become a major public health problem worldwide because of its high prevalence and because of the concomitant risk of metabolic diseases such as type 2 diabetes (T2DM)^9–11^. T2DM was the fourth leading cause of death of noncommunicable diseases worldwide in 2012^12^. In China, the prevalence of diabetes was 9.6% in 2013, corresponding to 100 million people^13^. A global systematic review revealed a large economic burden of diabetes, most directly affecting patients in many low- and middle-income countries in recent decades^14^.

With the rapid development of the economy, great changes in people’s lifestyle and dietary patterns in China have led to a high prevalence of chronic non-communicable diseases over the past decade^15^. Because of the large population and the great likelihood of impoverishment causes of chronic non-communicable diseases in rural areas, we need to investigate the current health conditions of rural dwellers and intervene in the occurrence and development of chronic non-communicable diseases in villages to achieve excellent social, economic, and health benefit. However, the density of health-care professionals is lower in rural than urban areas^16^. At present, the prevalence of HTGW among rural Chinese people is unknown, and data on the prevalence of T2DM among this population with HTGW is unclear. Despite a few studies on the association of HTGW and T2DM^17–19^, only one cohort study was from an urban Chinese adult population, and the association of transformation of the HTGW phenotype and risk of developing T2DM was not analyzed^11^. Therefore, we need to explore the potential relationship of HTGW and its dynamic status on the risk of developing T2DM in rural China.

In this cohort study, we investigated the incidence of HTGW and T2DM, the strength of the association of HTGW and T2DM risk, and the effect of HTGW transformation from baseline to follow-up on risk of developing T2DM among rural adult Chinese people.

## Results

### Demographic and clinical characteristics of study participants

Among the 12,086 participants at baseline, 37.22% (n = 4498) were males, and the median age was 50 years (interquartile range [IQR] 41–59). Baseline characteristics of participants, stratified by sex, are in Table 1. Females were younger and had higher BMI and triglycerides (TG), total cholesterol (TC), high-density lipoprotein cholesterol (HDL), and fasting plasma glucose levels as compared with males (*P* < 0.001). In addition, 587 males (13.05%) and 1887 (24.87%) females had HTGW, and males and females differed in distribution of “TG level and WC both normal”, “TG level and WC, only one being normal”, and “elevated TG level and enlarged WC” (HTGW) (*P* < 0.001).

**Table 1:** Baseline characteristics of study participants.

Characteristics	Males (n = 4498)	Females (n = 7588)	Total (n = 12,086)	*P* ^★^
Age (year; median [IQR])	53 (43, 61)	49 (41, 58)	51 (41, 59)	<0.001
WC (cm; median [IQR])	82.15 (75.35, 90.15)	81.50 (74.50, 88.60)	81.75 (74.90, 89.20)	<0.001
SBP (mmHg; median [IQR])	123 (113, 135)	122 (110, 138)	122 (111, 136)	0.008
DBP (mmHg; median [IQR])	77 (70, 85)	77 (70, 86)	77 (71, 86)	0.004
TG (mmol/L; median [IQR])	1.31 (0.95, 1.90)	1.39 (0.97, 2.01)	1.35 (0.96, 1.94)	<0.001
TC (mmol/L; median [IQR])	4.28 (3.74, 4.89)	4.46 (3.88, 5.12)	4.38 (3.82, 5.01)	<.0001
HDL (mmol/L; median [IQR])	1.09 (0.94, 1.26)	1.17 (1.02, 1.36)	1.14 (0.99, 1.32)	<.0001
FPG (mmol/L; median [IQR])	5.33 (4.97, 5.75)	5.37 (5.02, 5.82)	5.31 (4.97, 5.67)	<0.001
BMI (kg/m^2^; median [IQR])	23.28 (21.06, 25.68)	24.42 (21.98, 27.08)	24.05 (21.72, 26.54)	<0.001
BMI categories, kg/m^2^ (n [%])				<0.001
<24.0	2593 (57.65)	3376 (44.49)	5969 (49.39)	
24.0–27.9	1457 (32.39)	2887 (38.05)	4344 (35.94)	
≥28.0	448 (9.96)	1325 (17.46)	1773 (14.67)	
Cigarette smokers (n [%])	3236 (69.73)	17 (0.22)	3253 (26.61)	<0.001
Cigarette smoking (g/day; median [IQR]) *	15.0 (7.5, 15.0)	7.5 (3.0, 15.0)	15.0 (7.5, 15.0)	<0.001
Alcohol consumption (n [%])	1316 (28.36)	50 (0.66)	1366 (11.17)	<0.001
Alcohol consumption (ml/day; median [IQR]) *	22 (10, 50)	7 (3, 17)	20 (10, 50.0)	<0.001
Diabetic family history (n [%])	243 (5.24)	406 (5.35)	649 (5.31)	0.803
HTGW phenotype (n [%])				<0.001
NTNW	2573 (57.20)	2679 (35.31)	5252 (43.46)	
NTGW/HTNW	1338 (29.75)	3022 (39.83)	4360 (36.07)	
HTGW	587 (13.05)	1887 (24.87)	2474 (20.47)	

WC, waist circumference; SBP, systolic blood pressure; DBP, diastolic blood pressure; TG, triglycerides; TC, total cholesterol; HDL, high density lipoprotein cholesterol; FPG, fasting plasma glucose; IQR, interquartile range; *data for some participants were missing; NA, not available; ★comparison between different genders.

### Incidence of T2DM

**Figure 1:**
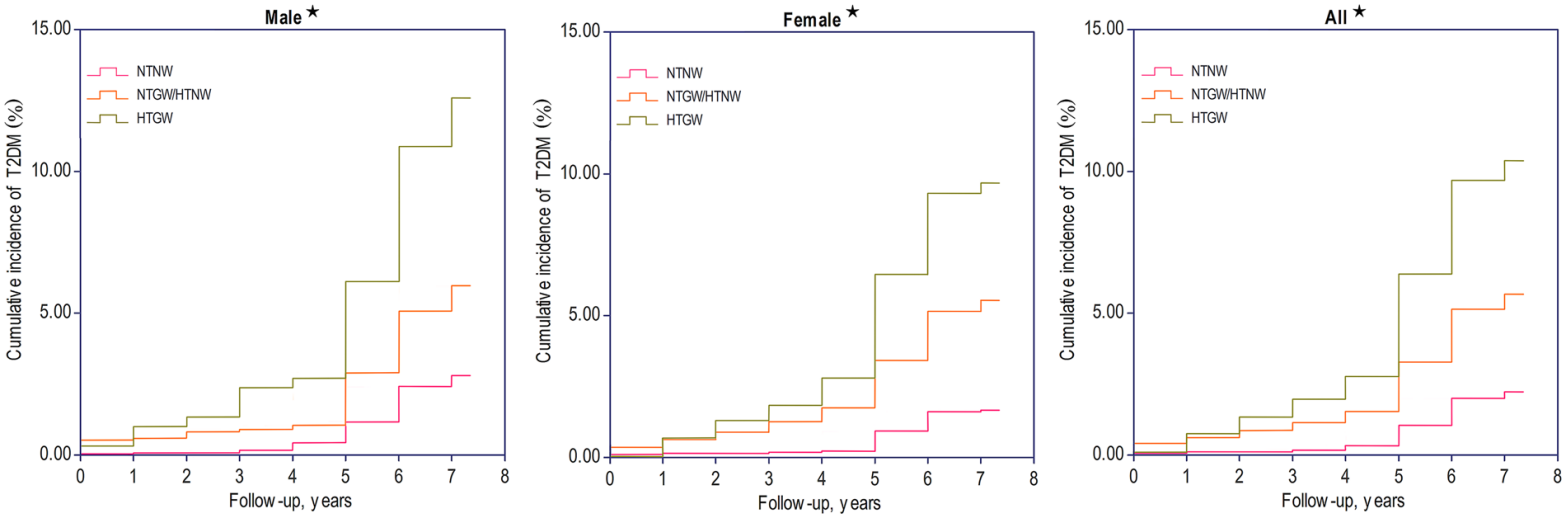
Cumulative incidence of T2DM for 3 phenotype groups. NTNW, normal triglycerides (TG) level (≤150 mg/dL [1.7 mmol/L]) and normal waist circumference (WC; <90 cm for males and <80 cm for females); NTGW, normal TG level and enlarged WC (≥90 cm for males and ≥80 cm for females); HTNW, elevated TG level (>150 mg/dL [1.7 mmol/L]) and normal WC; HTGW, elevated TG level and enlarged WC. ★, the cumulative incidence of T2DM is significantly different among NTNW, NTGW/HTNW, and HTGW (*P* < 0.001).

During a median follow-up of 6.0 years (range 0.06–7.10; 71,432 person-years of follow-up), T2DM developed in 621 participants (226 males). The cumulative incidence of T2DM stratified by the 3 phenotype groups for males, females, and overall are in Fig. 1. For HTGW participants, the incidence of T2DM was 26.4/1000, 20.6/1000, and 21.9/1000 person-years for males, females, and overall. The cumulative incidence of T2DM was significantly greater for HTGW than “TG level and WC, only one being normal” and “TG level and WC both normal” for males (12.61% vs. 5.98% vs. 2.80%), females (9.70% vs. 5.53% vs. 1.68%), and overall (10.39% vs. 5.67% vs. 2.23%) (all *P* < 0.001). Whether male or female, the cumulative incidence and incidence density of T2DM were higher for HTGW participants than those with “TG level and WC, only one being normal” and “TG level and WC both normal” (Fig. 1).

### Risk of developing T2DM by different transformations of HTGW phenotype

**Figure 2:**
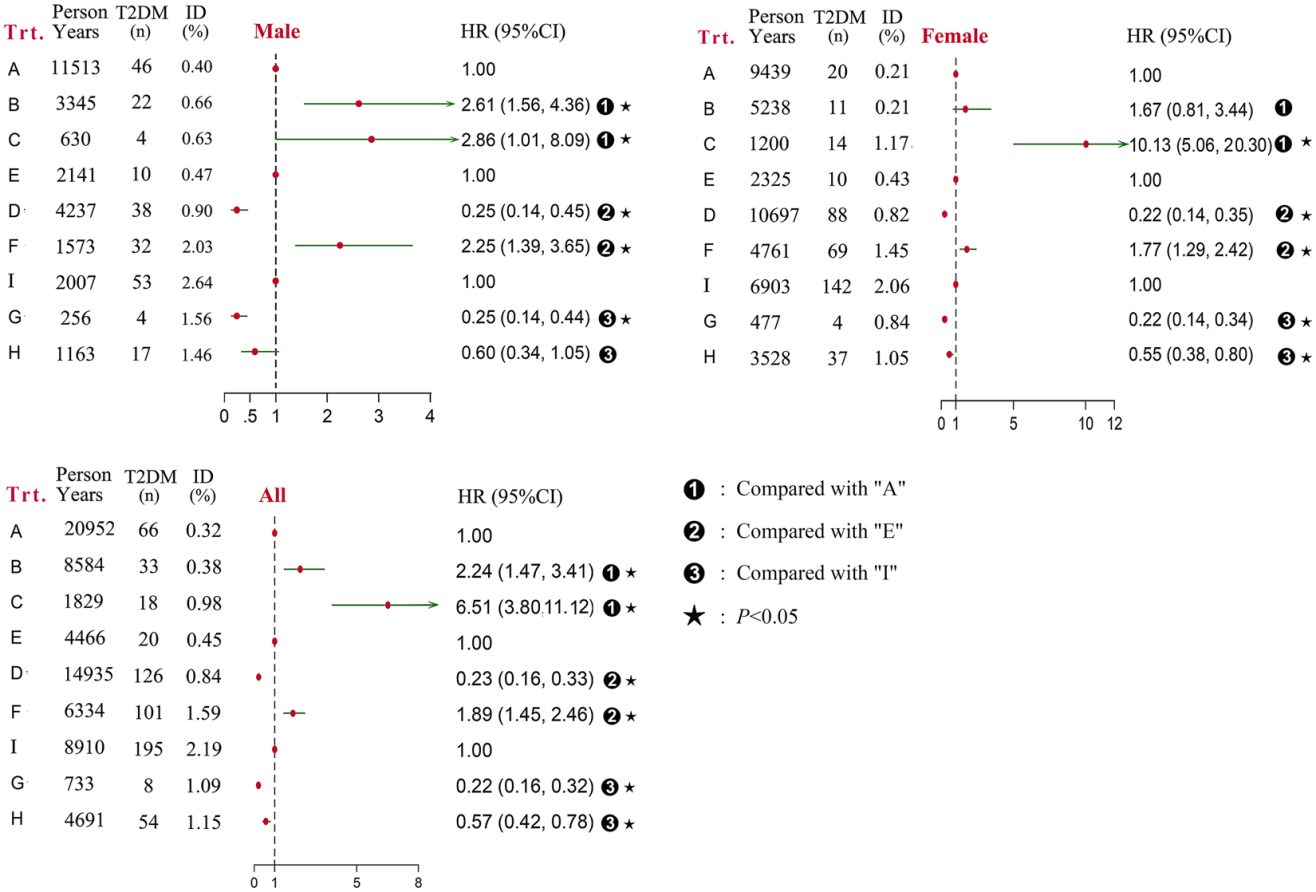
Risk of developing T2DM by different transformations of the 3 phenotype groups (Adjusted for sex, age, systolic blood pressure, diastolic blood pressure, smoking, alcohol drinking, diabetes family history). ID = Incidence density. Trt. = Transformation types. A = The consistent phenotype groups of “TG level and WC both normal” during follow-up. B = Baseline- “TG level and WC both normal” transformed to follow-up- “TG level and WC, only one being normal”). C = Baseline- “TG level and WC both normal” transformed to follow-up- “elevated TG level and enlarged WC”. D = Baseline- “TG level and WC, only one being normal” transformed to follow-up- “TG level and WC both normal”. E = The consistent phenotype groups of “TG level and WC, only one being abnormal” during follow-up. F = Baseline- “TG level and WC, only one being normal” transformed to follow-up- “elevated TG level and enlarged WC”. G = Baseline- “elevated TG level and enlarged WC” transformed to follow-up- “TG level and WC both normal”. H = Baseline- “elevated TG level and enlarged WC” transformed to follow-up- “TG level and WC, only one being normal”. I = The consistent phenotype groups of “elevated TG level and enlarged WC” during follow-up. elevated TG level and enlarged WC.

The multivariable-adjusted hazard ratios (HRs) for the risk of developing T2DM by transformation type after adjustment for sex, age, systolic blood pressure (SBP), diastolic blood pressure (DBP), smoking, alcohol drinking, BMI, and diabetic family history are in Fig. 2. Transformation types of the phenotype groups from baseline to follow-up are in Table 2. Types A, E, and I were the consistent phenotype groups (“TG level and WC both normal”, “TG level and WC, only one being normal”, and HTGW, respectively) during follow-up. Compared with “A” participants, for “C” participants (baseline “TG level and WC both normal” transformed to follow-up HTGW), the HR for T2DM was 2.86 (95% CI 1.01–8.09) for males and 10.13 (5.06–20.30) for females. For males, as compared with “A” participants, for “B” participants (baseline “TG level and WC both normal” transformed to follow-up “TG level and WC, only one being normal”), the relationship with T2DM remained significant (HR, 2.61; 1.56–4.36; *P* < 0.01). Compared with “E” participants, for “D” participants (baseline “TG level and WC, only one being normal” transformed to follow-up “TG level and WC both normal”), the HR for T2DM was favorable: 0.25 (0.14–0.45) for males and 0.22 (0.14–0.35) for females. For “F” participants (baseline “TG level and WC, only one being normal” transformed to follow-up HTGW), the HR for T2DM was 2.25 (1.39–3.65) for males and 1.77 (1.29–2.42) for females. Compared with “I” participants, except for male “H” participants (baseline HTGW transformed to follow-up “TG level and WC, only one being normal”), for both “G” (baseline HTGW transformed to follow-up “TG level and WC both normal”) and “H” participants, the HRs and 95% CI were both < 1.0. Overall, the results of Cox regression analysis for risk of developing T2DM by different transformations of HTGW phenotype demonstrated that the risk of HTGW for T2DM was reversible. As compared with the consistent phenotype groups, simply by recovering to normal TG level and/or normal WC, the risk of developing T2DM was greatly reduced (Fig. 2).

**Table 2:** Categories of transformations of the 3 phenotype groups between baseline and follow-up.

Phenotype groups		Transformation type
Baseline	Follow-up	
NTNW	NTNW	*A*
NTNW	NTGW/HTNW	B
NTNW	HTGW	C
NTGW/HTNW	NTNW	D
NTGW/HTNW	NTGW/HTNW	*E*
NTGW/HTNW	HTGW	F
HTGW	NTNW	G
HTGW	NTGW/HTNW	H
HTGW	HTGW	*I*

NTNW, normal triglycerides (TG) level (≤150 mg/dL [1.7 mmol/L]) and normal waist circumference (WC) (<90 cm for males and <80 cm for females); NTGW, normal TG level and enlarged WC (≥90 cm for males and ≥80 cm for females); HTNW, elevated TG level (>150 mg/dL [1.7 mmol/L]) and normal WC; HTGW, elevated TG level and enlarged WC.

### Impact of baseline phenotype groups on β-cell function and insulin sensitivity during follow-up

For both males and females, levels of fasting plasma glucose (FPG), fasting plasma insulin (FPI), homeostasis model assessment of insulin resistance (HOMA-IR), and HOMA of β-cell function (HOMA-β) were higher for patients with HTGW than “TG level and WC both normal” or “TG level and WC, only one being normal’ at baseline (*P* < 0.001) (Table 3). The increase in WC and TG level aggravated insulin resistance, which in turn increased insulin secretion to serve a compensatory function to maintain near-normal glucose tolerance. Nevertheless, pairwise comparison of HOMA-β between “TG level and WC, only one being normal” and HTGW groups showed no significant differences among females.

**Table 3:** Impact of baseline phenotype groups on β-cell function and insulin sensitivity during follow-up by sex.

Variables	Male		Female						All			
	NTNW	NTGW/HTNW	HTGW	P*	NTNW	NTGW/HTNW	HTGW	P*	NTNW	NTGW/HTNW	HTGW	P*
FPG	5.1 (4.7, 5.4)	5.2 (4.8, 5.7)	5.4 (4.9, 5.9)	<0.0001	5.0 (4.6, 5.4)	5.1 (4.7, 5.6)	5.3 (4.9, 5.8)	<0.0001	5.0 (4.7, 5.4)	5.2 (4.8, 5.6)	5.3 (4.9, 5.9)	<0.0001
FPI	8.6 (5.8, 11.8)	9.9 (6.9, 13.5)	12.0 (8.5, 16.8)	<0.0001	9.1 (6.2, 12.0)	10.3 (7.5, 14.1)	12.0 (8.6, 16.1)	<0.0001	8.8 (6.0,11.9)	10.2 (7.3, 14.0)	12.0 (8.6, 16.2)	<0.0001
HOMA-IR	1.9 (1.3, 2.7)	2.3 (1.6, 3.3)	2.9 (1.9, 4.1)	<0.0001	2.0 (1.4, 2.7)	2.3 (1.7, 3.3)	2.9 (2.0, 4.0)	<0.0001	2.0 (1.3, 2.7)	2.3 (1.6, 3.3)	2.9 (2.0, 4.0)	<0.0001
HOMA-β	112.0 (73.7, 168.0)	118.3 (77.3, 171.7)	130.2 (82.2, 190.1)	<0.0001	121.9 (82.0, 178.7)	128.9 (86.9, 186.0)	133.6 (88.4, 194.6)	<0.0001	117.3 (77.7, 174.4)	125.6 (83.9, 182.4)	132.8 (86.8, 193.6)	<0.0001

Data are median (interquartile range). FPG, fasting plasma glucose; FPI, fasting plasma insulin; HOMA-IR, homeostasis model assessment of insulin resistance; HOMA-β, homeostasis model assessment of β-cell function; NTNW, normal triglycerides (TG) level and normal waist circumference (WC); NTGW, normal TG level and enlarged WC; HTNW, elevated TG level and normal WC; HTGW, elevated TG level and enlarged WC. *Kruskal-Wallis test among NTNW, NTGW/HTNW, and HTGW.

### Risk of developing T2DM by the 3 consistent phenotype groups

**Figure 3:**
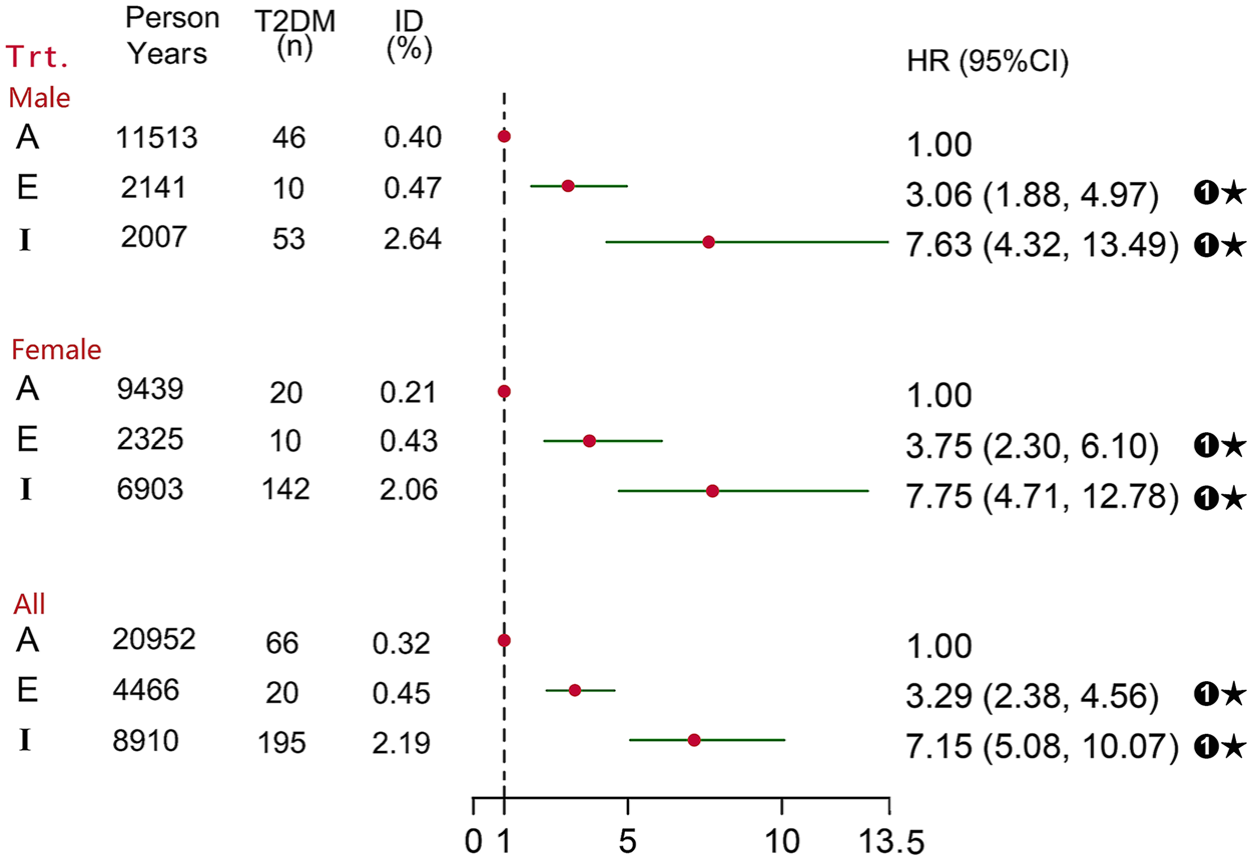
Risk of developing T2DM by the 3 consistent phenotype groups (Adjusted for sex, age, systolic blood pressure, diastolic blood pressure, smoking, alcohol drinking, diabetes family history). A = The consistent phenotype groups of “TG level and WC both normal” during follow-up; E = The consistent phenotype groups of “TG level and WC, only one being abnormal” during follow-up; I = The consistent phenotype groups of “elevated TG level and enlarged WC” during follow-up; ➊: compared with “A”; ★: *P* < 0.001.

In addition, we calculated the HR for risk of developing T2DM among consistent phenotype groups (Fig. 3). As compared with “A” participants, for “E” participants, the HR for T2DM was 3.06 (95% CI 1.88–4.97) for males and 3.75 (2.30–6.10) for females. Similarly, for “I” participants, the HR for T2DM was 7.63 (4.32–13.49) for males and 7.75 (4.71–12.78) for females. For “E” and “I” participants overall, the risk of developing T2DM was 3.29- and 7.15-fold greater, respectively, than for “A” participants. Hence, HTGW was a stable and significant metabolism type for T2DM risk.

## Discussion

The results of the present cohort study indicate that HTGW is a major risk metabolism type for T2DM in rural adult Chinese, yet the risk was temporarily reversible. After a median of 6.0 years’ follow-up, the cumulative incidence of T2DM with HTGW was 12.61% and 9.70% for males and females. As compared with “TG level and WC both normal”, with HTGW, the HOMA-IR index was higher and the risk of developing T2DM was 7.63- and 7.75-fold greater for males and females, respectively. Compared with consistent HTGW, with transformation from baseline HTGW to follow-up “TG level and WC both normal”, the risk of developing T2DM was reduced by 75% and 78% for males and females.

In a recent retrospective study of 2908 Chinese urban adults followed for 3 years, HTGW was associated with 3.77-fold increased risk of diabetes for males and 6.08-fold increased risk for females^13^, which agrees with our study and a study from Puerto Rico^11^. Okosun *et al*.^20^ reported a gender difference in the association of HTGW and T2DM. In the current study, stratification by sex revealed that the strength of the HTGW and T2DM association similar for both sexes but may differ in different ethnic and racial groups.

In our study, compared with WC and TG level at baseline, adverse transformation of one or both measures at the end of the follow-up was associated with significantly increased risk of developing T2DM; the transformation type associated with the greatest risk was baseline “TG level and WC both normal” transformed to follow-up HTGW for females, for increased risk of developing T2DM by 9.13-fold. In contrast, as compared with WC and TG level at baseline, transformation of one or both measures from abnormal to normal at the end of follow-up was associated with significantly decreased risk of developing T2DM; the most favorable transformation type was baseline HTGW transformed to follow-up “TG level and WC both normal” for females, which reduced the risk of developing T2DM by 78%.

A few studies have suggested HTGW as a screening and diagnostic predictor of T2DM^11, 13, 21, 22^, but no study has investigated the effect of these transformation types on risk of developing T2DM. Our study corroborates emerging evidence suggesting that the negative effect of abnormal metabolism on T2DM may be reversible: HTGW or “TG level and WC, only one being normal” returning to close to normal metabolic categories both had a positive effect on reducing the risk of developing T2DM.

In addition, with baseline “TG level and WC both normal” transformed to follow-up “TG level and WC, only one being normal”, males showed a greater risk of developing T2DM risk than females, but with baseline “TG level and WC both normal” transformed to follow-up HTGW, the risk was greater for females than males (HR 10.13 vs. 2.86), whereas males were insensitive to the transformation from baseline HTGW to follow-up “TG level and WC, only one being normal” (Fig. 2). This finding might be related to the different insulin metabolism between males and females^23^. HTGW is associated with increased insulin resistance and overstimulation of β-cell function^24^. Previous studies reported that females were more sensitive to insulin in terms of glucose metabolism than males^25–27^, so at the beginning of dysbolismus, the effect of insulin may offset part of the damage of T2DM in females. Once entering the decompensation period along with high susceptibility of insulin, the risk of developing T2DM would be higher for females than males. Therefore, the type of metabolic abnormality associated with T2DM, especially males with “TG level and WC, only one being normal” and females with “TG level and WC, only one being normal” or HTGW, should be a focus to prevent the premature development of T2DM, although the impact on HOMA-β between “TG level and WC, only one being normal” and HTGW at baseline did not differ among females.

The strengths of the study are primarily its prospective design and extensive follow-up (71,432 person-years). The metabolic type was based on measurement of TG plus WC at both baseline and follow-up, which effectively controlled the bias of the effect of transformation of different metabolic categories on the association of HTGW phenotype and T2DM, and the evaluation of the effect of the transformation of HTGW on risk of developing T2DM.

Although this was a relatively large sample cohort study, our study has some limitations. First, the transformation types of metabolic categories relied on measurement of TG plus WC at both baseline and follow-up, but we did not identify the reasons for transformation, lifestyle change or medical treatment, so development of targeted prevention efforts based on these data is difficult. Second, participants did not undergo an oral glucose tolerance test, which may underestimate the prevalence of diabetes. Third, because the participants were followed only once during the entire follow-up period, the measurement for change in TG level and WC for most participants with T2DM might be after the occurrence of T2DM, which could introduce information bias interfering in the true association. Finally, 14.5% of eligible participants were lost to follow-up and 21.5% of eligible participants had missing information on the presence of T2DM, which may be a source of bias and could be a major limitation of the study. Fortunately, the impact of these factors may be limited because the baseline characteristics of the participants followed up were similar to those without information regarding the presence of T2DM.

In conclusion, the present study documented that HTGW was a strong risk factor for T2DM. The risk of developing T2DM with abnormal metabolism was temporarily reversible. Strengthening programs and initiatives to prevent and control the HTGW phenotype should be a priority in national strategies to reduce the T2DM burden in China.

## Methods

### Study design and sample

A cohort of 20,194 participants age 18 to 92 years was recruited from rural areas of Henan Province, China, from July to August of 2007 and July to August of 2008. During baseline examinations, trained research staff used a standard questionnaire in an interview to assess demographic characteristics; lifestyle such as smoking, drinking, and medical family history of diseases; and other risk factors. After the interview, participants completed a physical examination that included evaluation of anthropometric indexes and blood pressure and collection of biological specimens. Body weight and height were measured twice to the nearest 0.5 kg and 0.5 cm, respectively, with the participant wearing lightweight clothing and no shoes. A single examiner used a standard measuring tape to measure WC twice at 1.0 cm horizontally above the navel over lightweight clothing to the nearest 0.5 cm with the participant in the standing position. Blood pressure was measured three times according to a standard protocol by using an electronic sphygmomanometer (Omron, HEM-770AFuzzy, Kyoto, Japan) with all participants in a sitting position after resting for 5 min. Overnight fasting blood samples were collected and stored at −20 °C, and a HITACHI automatic clinical analyzer (Hitachi 7060, Tokyo) was used for measuring FPG, TG, TC, HDL, and FPI. Insulin resistance and β-cell function were calculated from FPG and insulin data by the homeostasis model assessment of insulin resistance (HOMA-IR index)^28^: insulin resistance (HOMA-IR) = insulin/(22.5e^−ln glucose^), β-cell function (HOMA-β) = 20*insulin/(glucose-3.5).

A total of 17,262 participants among the 20,194 participants at baseline were successfully followed up from July to August of 2013 and July to October of 2014, with an overall response rate of 85.48%. The participants were followed once during entire follow-up period. Time-to-T2DM endpoint was based on the contents of the questionnaire or the fasting blood glucose levels at the endpoint of the follow-up. Recording of demographic characteristics, lifestyle, anthropometric indexes, blood pressure, and blood biochemical indicator detection was the same as for baseline examination. After excluding participants with underlying diabetes or with concurrent use of lipid-modified agents at baseline (n = 1857) and those without complete follow-up data for diabetes or HTGW ascertainment (n = 3319), 12,086 participants (7588 females) were retained for this analysis.

This study was approved by the Ethics Committee of Shenzhen University. All study participants provided written informed consent. All methods were performed in accordance with the relevant guidelines and regulations.

### Study variables

The values of body weight, height, WC, and blood pressure were the mean value of multiple measurements. Cigarette smoking was defined as having smoked ≥100 cigarettes during the lifetime^29^. Alcohol consumption was defined as consuming alcohol 12 or more times during the past year^29^. BMI was calculated as (weight in kg)/(height in m)^2^ and classified as normal weight (<24 kg/m^2^), overweight (24.0–27.9 kg/m^2^), and obesity (≥28 kg/m^2^)^30^. Hypertension was defined as mean SBP ≥140 mmHg and/or mean DBP ≥90 mmHg and/or use of antihypertensive medication within the past 2 weeks^31^. One or both parents having T2DM was considered a diabetic family history.

Participants were classified into 3 phenotype groups according to the International Diabetes Federation definition of metabolic syndrome for Chinese^32^ as follows: normal TG level (≤150 mg/dL [1.7 mmol/L])/normal WC (<90 cm for males and <80 cm for females); normal TG level/enlarged WC (≥90 cm for males and ≥80 cm for females) or elevated TG level (>150 mg/dL [1.7 mmol/L])/normal WC; HTGW: elevated TG level and enlarged WC. According to the transformation of the phenotypes between baseline and follow-up, 9 types were developed and are listed in Table 2.

### Definition of T2DM

According to the 2005 American Diabetes Association criteria^33^, T2DM was defined as fasting plasma glucose ≥7.0 mmol/L and/or the use of insulin or oral hypoglycemic agents and/or a self-reported history of diabetes with detailed diagnostic time and location, and without type 1 diabetes mellitus, gestational diabetes mellitus, or diabetes due to other causes.

### Statistical analysis

A cohort of 12,086 participants was retained for this analysis. Categorical data are represented as number (%) and were compared by chi-square test. Continuous data are represented as median (IQR) and were compared by Mann-Whitney-Wilcoxon test. The β-cell function and insulin sensitivity were compared among 3 groups by Kruskal-Wallis test, and the appropriate options in the MEANS statement in the ANOVA procedure of SAS 9.10 for Windows (SAS Inst., Cary, NC, USA) were used for multiple comparisons to compare the groups two by two. Person-years of follow-up were calculated from the date of the baseline questionnaire interview to the date of a T2DM event or follow-up interview for each participant, whichever came first. The cumulative incidence of T2DM for each phenotype group (based on TG level and WC at baseline) was analyzed by the Kaplan-Meier method, with differences in cumulative incidence of T2DM between different groups determined by Log rank test. The hazard ratios (HRs) of incident T2DM comparing transformation to consistent groups (based on TG level and WC at baseline and follow-up) were calculated for males and females by Cox proportional-hazard models. Among consistent groups, compared to “TG level and WC both normal”, the association of incident T2DM with HTGW or “TG level and WC, only one being normal” was calculated for males and females by Cox proportional-hazard models adjusted for age, smoking, alcohol drinking, medical treatment, SBP, DBP, BMI, and diabetic family history. Statistical analysis involved use of SAS 9.10 for Windows. P < 0.05 (two-sided) was considered statistically significant.
